# The impact of sporting event rituals on the wellbeing of Chinese university students

**DOI:** 10.3389/fpsyg.2025.1634076

**Published:** 2025-10-10

**Authors:** Qingfeng Liu, Yu Chen, Bing Liu

**Affiliations:** ^1^School of Economics and Management, Shanghai University of Sport, Shanghai, China; ^2^Physical Education Department, Shanghai University of Finance and Economics, Shanghai, China; ^3^Physical Education College, Shanghai University, Shanghai, China

**Keywords:** sporting event rituals, university students’ wellbeing, perceived social support, physical activity, Chinese higher education

## Abstract

**Background:**

As China ascends to become the world’s second-largest economy, its position at 68th in the 2024 Global Wellbeing Ranking underscores the persistence of the Easterlin Paradox. University students, who are expected to represent one of the most vibrant and promising social groups, are increasingly affected by mental health issues such as depression, self-harm, and even suicide, indicating that their sense of wellbeing remains insufficient and requires urgent attention. Some scholars argue that happiness is a form of capability that should be cultivated through public education interventions. As the fifth-largest component of China’s wellbeing economy, the sports industry, particularly sports events, which serve as a quintessential form of social ritual, plays a significant role in the pursuit of happiness. Building on this perspective, the present study proposes a theoretical model in which sports event rituals are conceptualized as the independent variable, university students’ wellbeing as the dependent variable, perceived social support as the mediating variable, and physical activity as the moderating variable.

**Methods:**

This study conducted a questionnaire survey among university students in Shanghai, yielding 1,256 valid responses. The data were analyzed using SPSS 26.0 and AMOS 24.0 to examine reliability and validity, as well as to test the hypothesized paths through regression analysis.

**Results:**

(1) Sporting event rituals have a significant positive effect on university students’ perceived wellbeing; (2) Perceived social support plays a significant mediating role in the relationship between sporting event rituals and students’ wellbeing; (3) Physical activity significantly moderates both pathways: it exerts a negative moderating effect on the relationship between sporting event rituals and wellbeing, and a positive moderating effect on the relationship between perceived social support and wellbeing.

**Conclusion:**

The findings contribute to a deeper understanding of the social role of sporting event rituals and provide a useful reference for addressing challenges in university sports education within developing country contexts.

## 1 Introduction

Wellbeing not only embodies individuals’ aspirations for a better life but also reflects a nation’s collective pursuit of social harmony and national prosperity. It is widely regarded as a fundamental goal of human development (Tan and Wu, 2014). However, the widely observed Easterlin Paradox, which posits that increases in national income do not necessarily lead to corresponding improvements in subjective wellbeing, has been repeatedly validated (Wei et al., 2018; Xia and Chen, 2022). In contrast, negative emotional states such as anxiety and emptiness have become increasingly prevalent in modern societies. In the context of China’s social development, the country has emerged as the world’s second-largest economy. Nevertheless, it ranks only 68th among 147 countries in the 2024 Global Happiness Index (Helliwell et al., 2024), indicating that China also faces the so-called “happiness–income paradox” (He and Pan, 2011). University students, as one of the most dynamic and promising demographic groups representing the hope of the nation’s future, play a critical role in shaping collective wellbeing. Although generally physically healthy, students at this transitional life stage are confronted with challenges such as academic pressure and interpersonal difficulties, both of which can significantly affect their mental health. According to the *Blue Book of Mental Health in China* (2022), the rates of depression and anxiety among university students have reached 18.5 and 30%, respectively, and continue to rise (Han et al., 2018; Li et al., 2020). In addition, feelings of loneliness, boredom, and social anxiety are widespread (Geng et al., 2025), reflecting a deeper psychological reality, namely, a pervasive sense of unhappiness among Chinese university students. Within the framework of positive psychology, developing students’ capacity for wellbeing has been recognized as a key strategy for addressing this issue. This approach emphasizes enhancing positive mental health as a means to reduce the incidence of psychological disorders (Seligman, 2018). As a central concept in positive psychology, wellbeing serves as a crucial indicator of collective mental health. Consequently, how to sustain or improve university students’ wellbeing has become a pressing concern not only in China but also globally (Lu, 2020).

Some scholars conceptualize wellbeing as a form of capability (Ekman and Simon-Thomas, 2021). For university students undergoing significant life transitions, developing this capability can not only help them cope with psychological difficulties but also facilitate smoother adaptation to post-graduation social life. In response, numerous universities worldwide, such as Harvard and Fudan, have introduced wellbeing courses aimed at cultivating students’ capacity for psychological resilience and emotional wellbeing. In addition to formal education, individual practices such as meditation or participation in social activities may also contribute to the improvement of wellbeing. Nevertheless, enhancing university students’ wellbeing remains a social issue that calls for more systematic and structured public educational interventions. Anthropological research suggests that human beings are inherently ritualistic, and ritual-based education serves as a powerful tool for shaping behavior. A growing body of literature indicates that family and school rituals significantly contribute to improving students’ subjective wellbeing (Jie and Liu, 2021; Neff and Germer, 2013). In recent years, the notion that “life needs ritual” has gained widespread popularity, reflecting the important role rituals play in the pursuit of happiness. While modern rituals have become increasingly secular and are often expressed through consumer or service-based experiences, many of these are driven by commercial interests. In contrast, sporting events, which are widely recognized as a form of social ritual (Birrell, 1981), carry cultural significance far beyond economic value. Through collective participation in competitive celebrations, symbolic narratives of victory, and emotionally immersive experiences, sporting events foster a sense of spiritual cohesion within communities—functions that were historically fulfilled by war or political ceremonies (Bell, 1991). In contemporary society, such events, characterized by inclusivity and interactive engagement, serve as emotional bridges that connect students with their families, schools, and the broader society. They resonate particularly well with the psychological needs of youth undergoing socialization and transformation.

To date, most research on sports events has focused on brand development, including brand personality building (Čáslavová and Petráčková, 2011), sport event innovativeness (Yoshida et al., 2013), Brand creation (Parent et al., 2012), and value co-creation (Grohs et al., 2020). Studies that examine sports events from the perspective of consumer perception have primarily concentrated on four dimensions: event image, brand awareness, event loyalty, and perceived value (Girish and Lee, 2019; Gholipour and Moradi, 2020; An and Yamashita, 2024). These dimensions are generally classified under functional value, emotional value, and relational value, indicating that value creation is central to the construction of sports events. However, there is a noticeable lack of attention to the non-commercial values embedded in sports events as social rituals, namely, the enduring sense of nobility, collective joy, and the symbolic satisfaction of citizens’ desires for a better life, social harmony, and national prosperity. Although some studies have explored how sports events contribute to individuals’ psychological resilience and adversity coping, few have systematically examined, from the perspective of ritual theory, the mechanisms by which these events enhance university students’ sense of social support and thereby improve their wellbeing. Consequently, the pathways and mechanisms through which sports events help students understand and improve their wellbeing remain insufficiently articulated. Against this backdrop, the present study investigates the impact of sports event rituals on the wellbeing of Chinese university students. By doing so, it not only provides a new analytical lens for addressing the global “development paradox,” but also seeks to draw on the traditional Chinese wisdom of “cultivating people through sports” to contribute a unique Chinese perspective to international discussions on student wellbeing.

## 2 Literature review and hypothesis generation

### 2.1 Sporting event rituals and university students’ wellbeing

“Ritual” has long been a critical area of inquiry in anthropology, sociology, and historiography. Its conceptual scope has gradually expanded from early mythological and religious practices to encompass various aspects of social life. To this day, the academic community has not reached a definitive consensus on “what constitutes a ritual.” Scholars have approached the concept from diverse disciplinary perspectives and methodological frameworks, resulting in a wide range of interpretations. Nevertheless, a general agreement has emerged around certain core features: rituals are structured, rule-bound behaviors characterized by procedural sequences, repeatability, and non-functionality. For instance, Turner et al.’s (2017) theory of liminality posits that rituals create a distinct spatiotemporal sphere through structured actions. Within this framework, sporting events, with their formalized procedures (e.g., opening and closing ceremonies, competitive sequences) and symbolic performances (e.g., songs, commentary, medals), align well with these three characteristics of ritual. In short, rural sports events can be regarded as a form of ritual, exhibiting the universal features commonly associated with ritual practices, such as spatial boundedness, symbolism, ethnic expression, and interactivity. The sense of ritual, or “ritual perception,” emerges in conjunction with ritual activities and is shaped by the specific events, settings, and behaviors in which individuals participate (Cui, 2012). Ritual is the external manifestation, while the sense of ritual is the internalized, affective response elicited through participation. Drawing on these insights, this study conceptualizes university students’ subjective perceptions of ritual expression in sports events encompassing five experiential dimensions: formality, ethnicity, interactivity, situationality, and symbolism. To emphasize this multidimensional perception, we refer to it throughout the paper as “sporting event rituals.”

Wellbeing, as a psychological construct, is closely linked to the culturally shaped development of the self-concept. The self-construal theory proposed by Markus et al. (1996) highlights the fundamental differences between Western and Eastern conceptualizations of the self: While Western cultures emphasize individualism and autonomy, Eastern cultures, particularly those influenced by Confucian values, such as China, tend to define the self in relation to others and the collective. Building on this perspective, Chinese scholars have argued that the Chinese understanding of happiness extends beyond individual life satisfaction to include harmonious interpersonal relationships and a sense of collective belonging at the national level (Lu and Shih, 1997; Gao et al., 2010). Accordingly, this study adopts the self-construal framework to conceptualize university students’ perceived wellbeing within China’s unique cultural context, and divides it into three dimensions: individual wellbeing, social wellbeing, and national wellbeing.

Existing studies on enhancing the wellbeing of university students have largely focused on individual-level factors such as motivational traits and cognitive beliefs. While these studies have demonstrated the importance of internal psychological mechanisms, they often overlook the social and cultural construction of psychological characteristics. In other words, although certain aspects of positive psychology are influenced by innate characteristics, they also require cultivation and guidance through broader public and social interventions during the university stage. From this perspective, some scholars have emphasized the role of social rituals in promoting wellbeing, particularly sporting event rituals. These events gather individuals around a shared focus of competition and, through collective expressions such as cheering and encouragement, help alleviate negative emotions and foster positive emotional experiences (Norton and Gino, 2014). In addition, the inherently interactive nature of sports events enhances emotional and verbal exchanges among students through shared attention on the competition and athlete performance, thereby improving the quality of social interaction (Hobson et al., 2018). Finally, sporting events often involve symbolic representations of the nation and ethnicity (Li, 2022). As participants, university students are likely to associate these symbols with national and cultural identity, thereby strengthening their sense of ethnic belonging, pride, and national honor through the ritual expressions of sports (Elling et al., 2014). Based on the above theoretical rationale, this study proposes the following hypothesis:

*H1:* Sporting event rituals positively influence university students’ perceived wellbeing.

### 2.2 The mediating role of perceived social support

Although some studies have examined the impact of sporting events on university students’ wellbeing, the mechanisms through which sports events, as ritualized experiences, affect wellbeing remain unclear. Therefore, it is necessary to explore the mediating role and facilitative mechanism of sporting event rituals in enhancing university students’ wellbeing. Perceived social support refers to an individual’s subjective perception of respect, support, and care from family, friends, and others (Zimet et al., 1988). During their developmental stage, university students are embedded in social interactions and networks; the closeness and quality of these social connections, as well as the corresponding level of social support, can significantly affect their negative emotions and mental health (Guo et al., 2023). According to social support theory, perceived social support plays a positive role in individual development (Lei et al., 2024); moreover, social influence theory suggests a strong association between social support and subjective wellbeing (Rachmad, 2025). Most studies have found that individuals with high levels of perceived social support tend to report higher levels of subjective wellbeing. Conversely, lower levels of perceived support are often associated with reduced wellbeing among university students. Research on the sources of perceived social support has shown that life rituals can serve as a channel through which university students experience a sense of social connectedness, thereby reducing feelings of loneliness (Guan, 2019).

Drawing on interaction ritual chain theory and social support theory, sporting events, as social rituals, provide participants and spectators with shared focal points for discussion and emotional expression. People from diverse backgrounds gather to engage in these events, generating social interactions that transcend boundaries (Ding, 2024). This kind of social integration and solidarity serves as a key source of perceived social support for university students (Inkelis et al., 2021). Through such social engagement, students can experience heightened feelings of social support, which in turn fosters a greater sense of internal security and contributes to wellbeing at the individual, social, and national levels. However, the extent to which perceived social support mediates the relationship between sporting event rituals and university students’ wellbeing, whether as a complete or partial mediator, remains inconclusive. Therefore, this study proposes the following preliminary hypothesis:

*H2:* Perceived social support plays a positive mediating role in the relationship between sporting event rituals and university students’ wellbeing.

### 2.3 The moderating role of physical activity

The Integrative Model of Human wellbeing emphasizes the impact of external factors (such as physical activity) on internal states (such as perceived wellbeing). Physical activity, as an external stimulus, is widely recognized for its role in improving individuals’ self-evaluations, thereby enhancing their overall wellbeing. Physical activity is any bodily movement produced by skeletal muscles that results in energy expenditure (Westerterp, 2013). Existing studies on the effects of physical activity on university students’ cognition and wellbeing have primarily focused on either physiological or psychological attributes in isolation. However, relatively few studies have examined how physical activity shapes students’ perceptions of their social environment, thus influencing their social attributes and wellbeing. Specifically, there is a lack of research investigating how different dimensions of physical activity, such as type, intensity, duration, and frequency, affect students’ social perception and subsequently contribute to their wellbeing.

According to the integrated psychological model proposed by Ryan and Deci (2001), individuals’ cognitive evaluations are influenced by their external environment, which in turn shapes various dimensions of perceived wellbeing (including individual wellbeing, social wellbeing, and national wellbeing). As a form of positive environmental stimulation, physical activity has been empirically shown to enhance individuals’ perceptions of ritual events and their sense of participation, thereby fostering a stronger sense of social connectedness (Davis et al., 2015). In this sense, students who engage in higher levels of physical activity tend to perceive sporting event rituals more intensely and report higher levels of perceived social support. Moreover, studies in exercise physiology have demonstrated that sustained physical activity facilitates the release of neurotransmitters such as endorphins, serotonin, and dopamine (Mammen and Faulkner, 2013), which are well known to improve mood and promote wellbeing and euphoria (Pilozzi et al., 2020). Thus, higher levels of physical activity are associated with a greater propensity for experiencing wellbeing. Taken together, physical activity, as an external factor, may play a moderating role in the relationships among sporting event rituals, perceived social support, and university students’ wellbeing. Based on this theoretical foundation, the following hypotheses are proposed:

*H3a:* Physical activity moderates the relationship between sporting event rituals and university students’ wellbeing.

*H3b:* Physical activity moderates the relationship between sporting event rituals and perceived social support.

*H3c:* Physical activity moderates the relationship between perceived social support and university students’ wellbeing.

## 3 Methods

### 3.1 Subjects and program

Given the current lack of empirical research on the model through which sports event rituals influence university students’ wellbeing, selecting a representative study population is particularly critical. This study focuses on undergraduate students from comprehensive universities in Shanghai, based on the following two main considerations: From the perspective of urban development representativeness, Shanghai is one of China’s most economically developed cities and a leading hub for sports events. It plays a significant radiating and demonstrative role in national urban development (Zhao, 2013) and is recognized globally as a prominent city for hosting major sports events. With a population of over 24 million, Shanghai exhibits intense social congestion. University students in this context face more complex pressures, including peer comparison, academic competition, and employment challenges. Notably, the prevalence of mental health issues among Shanghai university students has exceeded the national average for three consecutive years (Blue book of mental health, 2025), highlighting a generally low level of wellbeing. This phenomenon exemplifies the Easterlin paradox, where economic growth does not necessarily lead to increased happiness. Importantly, the contradiction between Shanghai’s rapid economic development and the relatively low wellbeing of its university students reflects trends and challenges that may emerge in other first-tier and even second- and third-tier cities across China in the future. Therefore, selecting Shanghai university students as the study population not only reflects the current development status of Chinese university students but also provides insight into future trends and potential issues in other major urban centers. From the perspective of exposure to sports events, the number and influence of such events have been growing rapidly across China, from cities to rural areas. Events like the “Jiangsu City Football League” and “Guizhou Rural Sports Games” illustrate the increasing social value and public engagement of sports activities. As one of China’s core cities for hosting large-scale sports events, Shanghai held over 9,100 domestic and international competitions annually, according to the Shanghai Municipal Sports Bureau in 2024. It gives Shanghai university students more frequent exposure to various sports event rituals, including opening and closing ceremonies, award ceremonies, and fan engagement activities, compared to their peers in other regions. Such high-frequency exposure increases their susceptibility to the cultural and psychological effects of event rituals, making them particularly suitable for testing the hypotheses of this study. In summary, selecting university students in Shanghai as the research sample not only provides valuable and practical insights for enhancing the quality of sports event organization in other cities from a ritual-based perspective but also contributes to the broader goal of improving the wellbeing of university students across China.

Considering these factors, this study was reviewed and approved by the Ethics Committee of Social Science Research at Shanghai University (Approval No: ECSSHU 2025-019). Data were collected using a convenience sampling method. An electronic questionnaire was distributed via the Credamo online survey platform to university students across universities in Shanghai. A total of 1,468 responses were received. After screening out invalid responses (e.g., identical answers, completion time <3 min, or patterned responses), 1,256 valid questionnaires were retained, yielding an effective response rate of 85.6%. Among the valid respondents, 829 were male (65.9%). The sample was mainly composed of first-year students (674, or 53.7%), followed by second-year students (385, or 30.7%). SPSS 26.0 was used to analyze, such as common method bias testing, descriptive statistics, and correlation analysis. Structural equation modeling (SEM) was performed using AMOS 24.0 to examine the mediating effect of perceived social support in the relationship between sporting event rituals and university students’ wellbeing across different levels of physical activity, and to derive the final research conclusions.

### 3.2 Research tools

#### 3.2.1 Sporting event rituals

This study adapted and revised the “Ritual Perception Scale” (Luo et al., 2024), originally developed in the context of tourism-related rituals in China, to measure university students’ perceptions of ritual expression in sports events. Considering the diversity of Sport event types and cultural contexts, the scale was designed to comprehensively capture the general characteristics of sports event rituals from the perspective of ritual universality. The scale aligns well with social ritual theory in terms of symbolic meaning, emotional involvement, and collective participation, making it suitable for contextual transfer to the domain of sports events. Minor contextual adjustments were made to better reflect the characteristics of sports event settings. Results from a pilot test indicated that all items were clearly worded and culturally appropriate.

The revised scale consisted of 23 items, including statements such as “Strictly enforced rules in sports events, such as referee whistles and foul judgments,” “During the sports event, I discuss or watch the games with friends or family,” and “This sports event fails to symbolize key social values such as fairness, justice, and integrity,” featuring both positively and negatively worded items. After reverse scoring and score transformation, higher total scores reflected a higher perceived level of ritual in sports events. Before the formal survey, a pilot test was conducted with 300 participants. Confirmatory factor analysis (CFA) results indicated good model fit: *x*^2^/df = 1.81, RMSEA = 0.041, TLI = 0.974, GFI = 0.971, with Cronbach’s *α* values for the five dimensions reported as 0.885, 0.915, 0.880, 0.857, and 0.881 respectively, demonstrating satisfactory reliability and validity. In the formal survey, CFA again confirmed the robustness of the scale: *x*^2^/df = 4.38, RMSEA = 0.052, TLI = 0.958, GFI = 0.957, AGFI = 0.929, and an overall Cronbach’s *α* of 0.924.

#### 3.2.2 Perceived social support

Perceived social support was measured using the 12-item Perceived Social Support Scale developed by Zimet et al. (1988) and revised by Chinese scholars Jiang et al. (1999). Sample items include statements such as “I can share my joys and sorrows with certain people (relatives/classmates/teachers, etc.),” “I can talk to my family about my problems,” and “When things go wrong, I feel I cannot count on my friends,” with both positively and negatively worded items included. After score conversion, higher total scores indicate a higher level of perceived social support. The results of confirmatory factor analysis (CFA) indicated a good model fit: *x*^2^/df = 3.26, RMSEA = 0.042, TLI = 0.989, GFI = 0.985, AGFI = 0.970, the internal consistency of the scale was acceptable, with Cronbach’s *α* of 0.817.

#### 3.2.3 University students’ wellbeing

There are currently 69 validated scales for measuring individual wellbeing (Zhang et al., 2024), covering nine domains of wellbeing, including gratitude, social relationships, and more. This diversity reflects the conceptual complexity of individual wellbeing and indicates that different populations often require culturally adapted measurement tools. In the context of Chinese university students, who are influenced by China’s collectivist cultural traditions, the perception of wellbeing extends beyond the individual level to include social and national dimensions. Therefore, this study employs three well-established scales to measure the multidimensional nature of wellbeing: the Subjective Wellbeing Scale (Zhang and Carciofo, 2021) for individual wellbeing, the Relationship Assessment Scale (Ryff, 2013) for social wellbeing, and the Ethnic Identity Scale (Phinney, 1992) for national wellbeing.

The composite wellbeing scale included 15 items, such as “This sport event improves the quality of my life,” “This event adds joy to my time with family and friends,” and “This event presents a positive image of China, but I did not feel proud”—with both positively and negatively worded items. After reverse scoring where necessary, higher total scores indicated higher levels of wellbeing among Chinese university students. Prior to the main survey, a pilot study was conducted with 300 participants. Results from confirmatory factor analysis (CFA) showed good model fit: *x*^2^/df = 1.23, RMSEA = 0.054, TLI = 0.947, GFI = 0.905, and Cronbach’s α coefficients of 0.776, 0.762, and 0.704 for the three subscales, indicating satisfactory reliability and validity. In the formal survey, CFA results further confirmed the robustness of the scale: *x*^2^/df = 2.64, RMSEA = 0.036, TLI = 0.993, GFI = 0.994, AGFI = 0.981, and an overall Cronbach’s *α* of 0.915.

#### 3.2.4 Physical activity

This study measured university students’ physical activity levels using the *International Physical Activity Questionnaire – Short Form* (IPAQ-SF) (International Physical Activity Questionnaire - Short Form, 2025). The questionnaire consists of seven items assessing participants’ physical activity over the past week, focusing on *exercise frequency* and *duration*. The overall activity level score was calculated using the following formula: Physical activity score = exercise frequency (days/week) × exercise duration (minutes/day) × metabolic equivalent of task (MET) value corresponding to the activity intensity. Based on established criteria (Qiao, 2017), participants’ physical activity levels were classified into three categories: low physical activity, medium physical activity, and high physical activity. Chinese scholars Li and Li (2020) tested the questionnaire’s validity using three different methods, and the localized version of the IPAQ-SF demonstrated good reliability and validity in the Chinese context.

## 4 Results

### 4.1 Common method biases test

Data were collected through a self-report questionnaire, which may introduce the risk of common method bias. To assess this potential issue, we adopted the commonly used diagnostic approach for detecting such bias in self-reported surveys (Xiao et al., 2025; Yuan et al., 2025). Specifically, Harman’s single-factor test was conducted. The results showed that the variance explained by the first unrotated factor was 12.72%, which is well below the critical threshold of 40%. It indicates that common method bias is within an acceptable range, and the data are suitable for subsequent analysis.

### 4.2 Descriptive statistics of university students with different physical activity levels

As shown in Table 1, among the participants in the past week, 328 students (26.1%) reported a low level of physical activity, 432 students (34.4%) reported a moderate level, and 496 students (39.5%) reported a high level of physical activity.

**Table 1:** Descriptive statistics of different physical activity levels (*N* = 1,256).

Physical activity level	Frequency	Percentage (%)
Low physical activity	328	26.1
Medium physical activity	432	34.4
High physical activity	496	39.5

### 4.3 Correlation analysis and *AVE* result

Based on the correlation coefficients and validity measurement results of each dimension (Table 2), significant positive correlations (*p* < 0.001) were found among the 11 dimensions under the variables of sports event ritual, perceived social support, and university students’ wellbeing. The bold diagonal values in Table 2 represent the square roots of the *AVE* (Average Variance Extracted), all of which are greater than the correlation coefficients in their respective rows and columns. This indicates strong discriminant validity among the constructs. For instance, as highlighted in the shaded section of Table 2, the square root of the *AVE* for the variable “other support” is 0.695, which exceeds all corresponding correlation coefficients in its row and column, confirming the discriminant validity of this variable. In addition, the composite reliability (*CR*) values for all constructs exceed the recommended threshold of 0.7, and the *AVE* values are all above or close to the critical value of 0.5. These results collectively demonstrate that the measurement scales used in this study exhibit good reliability and validity.

**Table 2:** Correlation coefficients and validity measurement between dimensions (*N* = 1,256).

	(1)	(2)	(3)	(4)	(5)	(6)	(7)	(8)	(9)	(10)	(11)
(1) Formality	**0.737** ^**a**^										
(2) Ethnicity	0.578^b^**	**0.846**									
(3) Interactivity	0.524**	0.441**	**0.709**								
(4) Situationality	0.465**	0.330**	0.541**	**0.815**							
(5) Symbolism	0.556**	0.627**	0.475**	0.454**	**0.677**						
(6) FRS	0.288**	0.231**	0.400**	0.311**	0.260**	**0.854**					
(7) OS	0.316**	0.056*	0.361**	0.361**	0.191**	0.509**	**0.695**				
(8) FAS	0.308**	0.419**	0.436**	0.328**	0.299**	0.528**	0.291**	**0.854**			
(9) IW	0.451**	0.409**	0.510**	0.652**	0.512**	0.451**	0.415**	0.441**	**0.801**		
(10) SW	0.452**	0.314**	0.514**	0.576**	0.437**	0.489**	0.424**	0.413**	0.742**	**0.817**	
(11) NW	0.335**	0.601**	0.334**	0.343**	0.601**	0.398**	0.113**	0.346**	0.527**	0.445**	**0.853**
CR	0.825	0.909	0.8	0.856	0.808	0.915	0.789	0.915	0.909	0.909	0.914
AVE	0.543	0.716	0.502	0.665	0.459	0.729	0.483	0.73	0.642	0.667	0.728

**p < 0.01; ^a^The bold values on the diagonal of the correlation matrix represent the square roots of the AVE (Average Variance Extracted); ^b^The lower-left triangle of the correlation matrix displays the correlation coefficients. Bold values indicate the square root of the AVE. PA, physical activity; FRS, friend support; FAS, family support; IW, individual wellbeing; SW, social wellbeing; NW, national wellbeing.

### 4.4 ANOVA of variables across different levels of physical activity

As shown in Table 3, significant differences were found in university students’ wellbeing, sports event rituals, and perceived social support across different levels of physical activity, indicating that physical activity has a significant impact on sports event rituals, perceived social support, and university students’ wellbeing.

**Table 3:** Analysis of variance among different physical activity levels for each variable.

Dependent variable	(I) PA	(J) PA	Mean Different (I-J)	Boot SE.	Boot LLC	Boot ULCI
CSW	Low	Medium	−0.270*	0.065	−0.397	−0.143
	Low	High	−0.752*	0.063	−0.876	−0.629
	Medium	High	−0.482*	0.058	−0.597	−0.368
SER	Low	Medium	−0.371*	0.055	−0.479	−0.263
	Low	High	−0.543*	0.053	−0.648	−0.438
	Medium	High	−0.172*	0.049	−0.269	−0.075
PSS	Low	Medium	−0.168*	0.067	−0.300	−0.037
	Low	High	−0.510*	0.065	−0.638	−0.382
	Medium	High	−0.342*	0.060	−0.460	−0.223

^*^p < 0.05; SER, sporting event ritual; PSS, perceived social support; CSW, college student wellbeing.

### 4.5 Path analysis

First, the regression analysis revealed that sports event rituals had a significant positive predictive effect on university students’ wellbeing (β=0.546, p<0.001), supporting Hypothesis 1 (H1). Second, perceived social support also significantly predicted university students’ wellbeing (β=0.319, p<0.001), and sports event rituals had a significant positive predictive effect on perceived social support (β=0.48, p<0.001). Therefore, perceived social support played a partial mediating role between sports event rituals and university students’ wellbeing. The mediating effect was further confirmed by the Bootstrap test (p<0.001), thus supporting Hypothesis 2 (H2).

**Figure 1:**
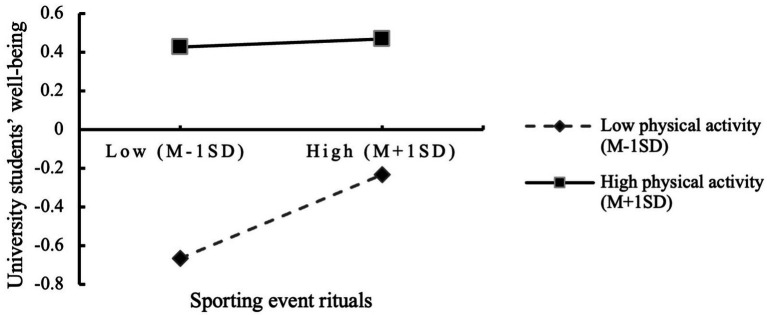
The moderating effect of physical activity on the relationship between sporting event rituals and university students’ wellbeing.

**Figure 2:**
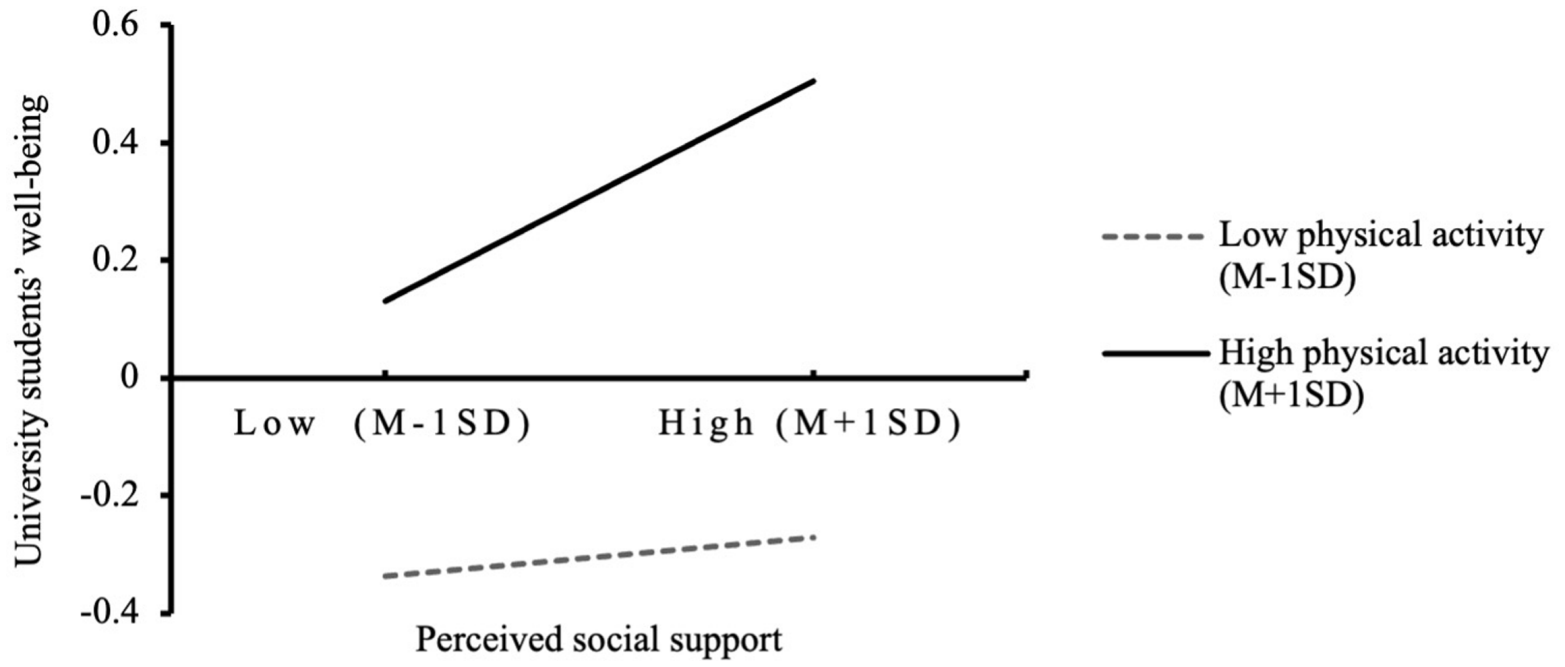
The moderating effect of physical activity on perceived social support and university students’ wellbeing.

**Figure 3:**
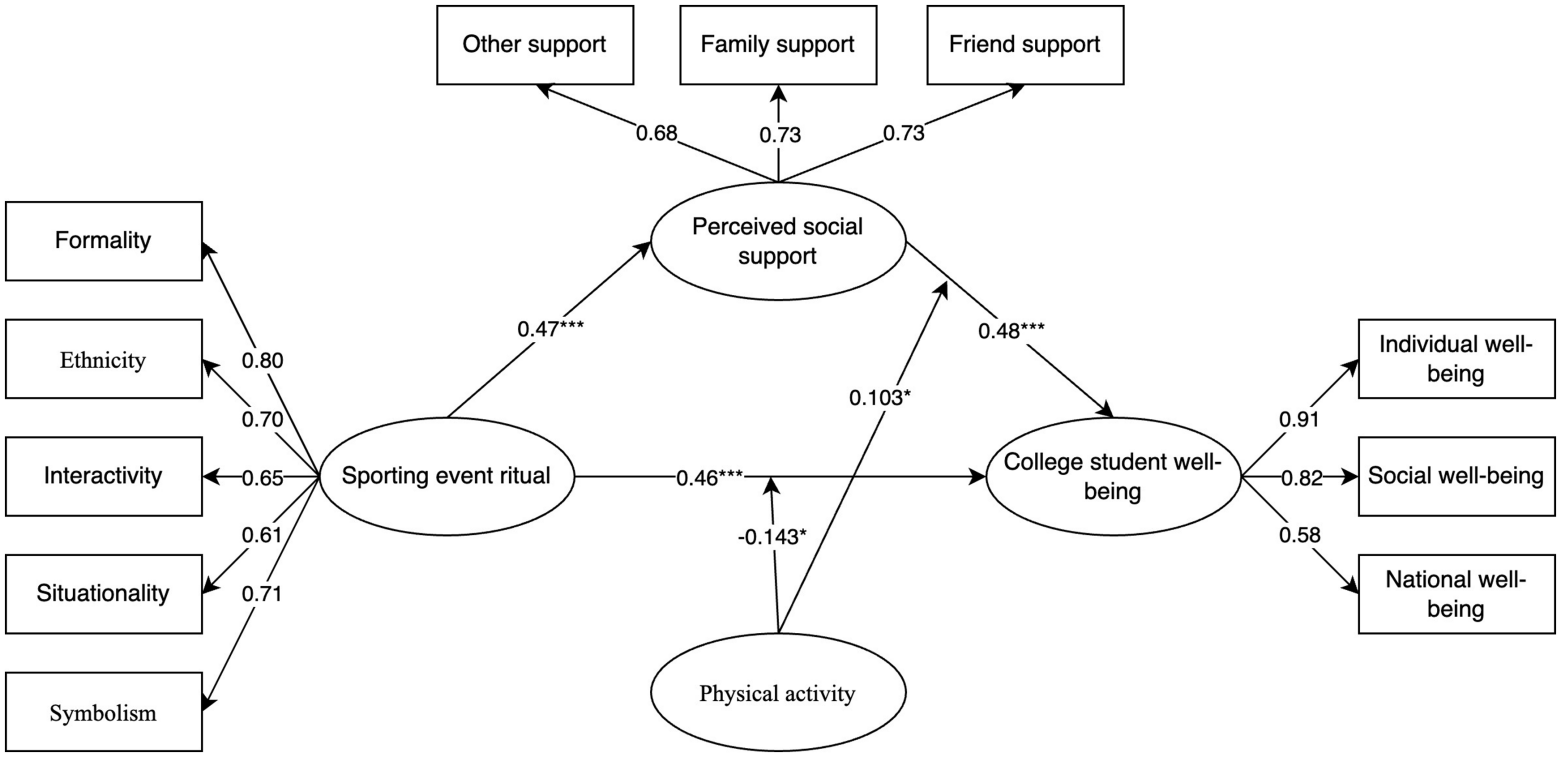
Structural equation model diagram.

Next, to test the moderating role of physical activity, all variables were mean-centered. Using PROCESS regression analysis with moderate physical activity as the reference group, it was found that physical activity did not significantly moderate the relationship between sports event rituals and perceived social support (p=0.175), and thus Hypothesis 3b (H3b) was not supported. However, physical activity significantly moderated the relationship between sports event rituals and university students’ wellbeing, as well as between perceived social support and university students’ wellbeing. As shown in the simple slope plots (see Figures 1, 2), physical activity exerted a significant positive moderating effect on both relationships. Accordingly, the final model of this study is illustrated in Figure 3.

## 5 Discussion

### 5.1 The relationship between sporting event rituals, perceived social support, and university students’ wellbeing

Enhancing university students’ wellbeing has become a key concern in China’s higher education system. In recent years, scholars have sought to improve students’ wellbeing through the environmental–psychological cognition framework and have identified rituals as a critical public mechanism for enhancing wellbeing (Martin et al., 2015). However, whether sports events, when regarded as ritualized experiences, play a pivotal role in promoting university students’ wellbeing remains unclear. To address this gap, this study investigates the influence of sports event rituals on students’ perceived wellbeing from a cognitive perspective. Regression and structural equation modeling results indicate that sports event rituals significantly and positively predict university students’ wellbeing, supporting Hypothesis 1. The finding corroborates the classical proposition that “rituals are an important source of happiness” (Kumar, 2013) and further validates the applicability of Collins’ (2014) interaction ritual chain theory in the domain of sport. That is, the symbolic systems and interaction processes embedded within sports event rituals can effectively activate emotional energy and facilitate sense-making. Moreover, this study offers a counterpoint to the growing phenomenon of “pseudo-ritual” among Chinese university students. For instance, the so-called ritual of buying “the first cup of milk tea in autumn” has become a commodified social gesture among young people. However, Zhao and Feng (2022) argue that students’ focus on such superficial rituals neglects the emotional transmission and symbolic meaning inherent in authentic ritual experiences. This trend may reflect a deeper emotional void among contemporary Chinese youth, who seek wellbeing through ritualistic behaviors. By contrast, sports event rituals, with their intrinsic symbolic structures (e.g., trophies, theme songs, narratives of competitive spirit) and ritualized actions (e.g., victory cheers, celebratory gestures), provide a framework for meaning construction that transcends individual experience. According to sense-making theory, collective rituals shaped by symbolic systems and interpersonal interaction help students construct deeper meaning connections at the personal, social, and even national level, thereby fostering more sustained wellbeing.

This study also confirms the significant mediating role of perceived social support in the relationship between sports event rituals and students’ wellbeing. Hypothesis 2 is supported. In addition to its direct effect, sports event ritual participation indirectly enhances students’ wellbeing by increasing their perceived social support. Previous studies have shown that perceived social support is a critical determinant of psychological adjustment and university students’ wellbeing. According to self-determination theory, individuals have three basic psychological needs: competence, relatedness, and autonomy. Perceived social support, as a response to the need for relatedness, is essential for holistic psychological development (Tan et al., 2017). High-level perceived support can generate positive psychological energy contributing to personal growth (Pan et al., 2017), enabling students to cope more effectively with academic and life stressors. However, the mechanisms by which perceived social support mediates wellbeing outcomes have not been thoroughly explored, particularly regarding its sources and dynamic functions among university students. While some studies have proposed psychological interventions to enhance perceived support, they have primarily confirmed its importance without offering valid strategies for improvement or clarifying how it mediates wellbeing. This limitation has led to unsustainable practices in Chinese universities. For example, although students are encouraged to engage in volunteer service to build social connections, these activities are not embedded in the core curriculum and often lack sufficient planning. Similarly, while psychological counseling services and student employment programs reflect institutional concern for students’ needs, the growing incidence of depression, self-harm, and suicide among university students suggests that current initiatives are insufficient in fostering meaningful perceptions of support.

The findings of this study provide empirical support for social integration theory, which posits that collective rituals reinforce social norms and group identity, thereby enhancing individual happiness. As a typical form of social ritual, sports events create a symbolic environment (e.g., team logos, slogans, ceremonial procedures) as a focal point for group interaction. Through shared attention, coordinated action, and emotional resonance, participants forge meaningful connections that transcend the individual (Cottingham, 2012). This ritual-driven integration process not only strengthens students’ identification with the event and sense of belonging but also transforms abstract notions of social support into embodied emotional connections. In turn, this deepens students’ holistic understanding of both personal and collective wellbeing. This mechanism also reinforces the educational value of university sports events, when structured with ritualized frameworks, these events transcend mere competition and serve as dynamic platforms for constructing social support networks among students.

It is worth noting that the results of this study align with Luo and Mu’s (2017) research on high school students, which found that the effect of perceived social support on psychological wellbeing was fully mediated by the satisfaction of basic psychological needs. In contrast, our study found both direct and mediating effects of perceived support, which may reflect developmental differences in the two samples (Wigfield et al., 1996). Compared with high school students, university students have more mature cognitive abilities and can derive support directly from group interaction during event participation, whereas adolescents may rely more on indirect mechanisms linked to need fulfillment. This insight highlights the importance of adopting a life-course perspective in future research on social support processes and designing phase-specific intervention strategies accordingly.

### 5.2 The moderating role of physical activity

Previous research has often treated physical activity as a buffer against psychological problems—for instance, alleviating symptoms of depression (Xu et al., 2023) or reducing perceived stress (Awick et al., 2017). In contrast, this study explores the facilitative moderating role of physical activity in the mechanism through which Chinese university students perceive wellbeing. Our findings reveal that physical activity, as a moderating variable, exhibits significant context-dependent effects. Specifically, physical activity weakens the impact of sports event rituals on wellbeing while enhancing the effect of perceived social support, reflecting its multifaceted function as a behavioral resource. The moderating pathways of physical activity in this study are discussed below:

First, physical activity did not significantly moderate the relationship between sports event rituals and perceived social support, and thus, Hypothesis 3a was not supported. One possible explanation is that perceived social support is fundamentally the result of interactions between individuals and their microsystems (Shuo et al., 2022), while physical activity, as a measure of individual behavioral engagement, functions at a different level than the interactional processes central to rituals. The influence of sports event rituals on perceived social support largely depends on the quality of interpersonal interaction, such as shared attention, collaborative engagement, and emotional resonance. Among students with high levels of physical activity (78.3% of whom in our sample identified as regular exercisers), there tends to be a greater focus on athletic performance during the event, often at the expense of attending to social interactions embedded in the ritual context. It aligns with the theory of limited attentional capacity: when individuals concentrate on one type of information, they are more likely to overlook others (Van Knippenberg et al., 2015). Conversely, students with lower physical activity levels may gain more perceived social support through symbolic group interactions such as cheering and collaboration. This finding underscores the symbolic nature of sports events as collective rituals—their influence on perceived social support stems from symbolic group connection rather than the physicality of individual participation. It also supports social integration theory, which argues that collective rituals enhance perceived support through shared meaning and emotional bonding. In such highly symbolic contexts, individual physiological differences may be attenuated.

Second, physical activity demonstrated a significant negative moderating effect on the relationship between sports event rituals and university students’ wellbeing, supporting Hypothesis H3b. This finding contrasts with the results of Shanshan et al. (2025), where physical activity positively moderated the relationship between physical fitness and wellbeing. The discrepancy may stem from the differing functional relationships between variables: in Shanshan et al. (2025)’s study, physical activity interacted with physical fitness in a mutually reinforcing manner, whereas in the current study, sports event rituals and physical activity may exhibit some psychological substitutability. Specifically, two underlying mechanisms may account for this effect. On the one hand, individuals with higher physical activity levels tend to report greater baseline wellbeing, as regular exercise promotes the release of endorphins and enhances self-efficacy (Brellenthin et al., 2017; Moritz et al., 2000). Their fundamental psychological needs may already be satisfied through physical activity, thereby diminishing the “added value” of ritual participation. According to self-determination theory, when individuals’ autonomy needs are fulfilled in other contexts, the controlling cues embedded in external rituals may suppress intrinsic motivation and, in turn, reduce the contribution of rituals to wellbeing. In contrast, individuals with lower levels of physical activity may be more inclined to attend to the symbolic elements of sports events and emotionally engage with others during participation, thus experiencing greater psychological benefits from the ritualized atmosphere. On the other hand, cognitive differences related to physical activity levels may also play a role. According to the theory of attentional resource allocation (Kahneman, 1973), cognitive capacity is a limited psychological resource. Compared with their low-activity counterparts, students with higher physical activity levels tend to focus more on the technical and competitive aspects of sports events (Gao et al., 2021) while paying relatively less attention to the symbolic meaning of rituals. In contrast, those with lower physical activity levels may place greater emphasis on ritualistic expressions and symbolic content. These differences in cognitive orientation may help explain why the psychological empowerment effect of sports event rituals weakens as physical activity levels increase.

Third, physical activity significantly and positively moderated the relationship between perceived social support and wellbeing, supporting Hypothesis 3c. It suggests a synergistic effect between perceived social support and physical activity. Among students with high levels of physical activity, perceived social support had the strongest positive predictive effect on wellbeing. It aligns with the theory of resource synergy, which posits that individuals with higher physical activity levels possess stronger social interaction and emotional regulation capacities (Liu et al., 2023; Wang et al., 2020). Compared to both low and high levels of support among low-activity individuals, students with high physical activity demonstrated significantly higher levels of perceived social support (p<0.05), and were more effective in translating that support into subjective wellbeing. Although previous studies have not always distinguished the influence of different physical activity levels on the perception of social support or its predictive power for wellbeing, existing literature consistently indicates that active engagement in physical activity promotes both perceived support and wellbeing (Sui et al., 2024; Yuan and You, 2022), this study clarifies the logic connecting these three elements, showing that students with different physical activity levels respond differently to perceived support in terms of wellbeing outcomes.

These findings suggest that educators should adopt differentiated approaches based on students’ physical activity levels. While increasing overall physical activity among students should remain a strategic priority for universities, given its benefits for enhancing social support and promoting dopamine secretion, thereby improving subjective wellbeing (Liao et al., 2023), targeted interventions may be necessary. For students with low physical activity levels, incorporating light forms of group exercise (e.g., cheerleading routines, collective dances) and framing them with ritualistic significance may help reduce negative associations and transition students toward sustained physical activity habits. For highly active students, educational strategies should emphasize the symbolic and emotional dimensions of sports event rituals. For example, interactive pre-event rituals could be designed to enhance emotional resonance and social bonding with other participants, thereby reinforcing the sense of ritual and its connection to wellbeing.

## 6 Conclusion

This study developed a theoretical model integrating sports event rituals, university students’ wellbeing, perceived social support, and physical activity. The results reveal that sports event rituals enhance students’ wellbeing at three levels, personal, social, and national, by fostering their ability to perceive social support. Physical activity plays differential moderating roles within this model: it negatively moderates the relationship between sports event rituals and wellbeing, while positively moderating the relationship between perceived social support and wellbeing. These findings suggest that, in addition to promoting overall physical activity among university students, tailored strategies should be implemented based on students’ varying levels of physical activity. This study deepens our understanding of the social value of sport by framing it within a societal perspective. It also contributes to bridging the gap between sport-related research and broader social issues, offering valuable insights for scholars seeking to address societal challenges through sport-based interventions.

### 6.1 Theoretical implications

First, this study addresses the widely relevant social issue of university students’ wellbeing by exploring the role of sport in promoting individual wellbeing. It emphasizes that research on the value of sport should serve pressing societal issues such as social development and mental health. Specifically, from the perspective of ritual education, the study reveals the positive function of ritual expressions embedded in sports events in enhancing students’ wellbeing. Furthermore, from an environmental perspective, it examines the potential moderating role of physical activity as a positive external factor in the relationship between sports rituals and psychological wellbeing, thereby enriching the theoretical framework at the intersection of sport and psychology.

Second, this study extends the application of ritual-related theories to the fields of sport and psychology. By integrating social identity theory and interaction ritual chain theory, it constructs a theoretical model to explain how sports event rituals influence university students’ wellbeing. The empirical validation of this model supports its theoretical applicability and promotes the expansion of ritual theory into the domain of mental health in higher education. This theoretical extension offers valuable references for future studies in diverse cultural and sports contexts.

Finally, from a methodological perspective, this study systematically examines the mechanisms through which sports event rituals affect students’ wellbeing and clarifies the mediating and moderating roles of perceived social support and physical activity. These findings contribute to a more nuanced understanding of how sport-based interventions can influence youth mental health. The proposed theoretical model deepens academic comprehension of the underlying mechanisms linking sports event rituals and psychological wellbeing, while also providing theoretical support for practical interventions in this area.

### 6.2 Practical implications

Our findings provide targeted, practical guidance for designing and implementing sports event rituals, particularly in enhancing university students’ wellbeing.

First, the results demonstrate that the ritual expression of sports events plays a positive role in promoting university students’ wellbeing, providing valuable direction for physical education professionals, event organizers, and university administrators to improve event planning and execution. It is recommended to emphasize ritual features in event design, such as incorporating symbolic opening and closing ceremonies, props, slogans, and interactive elements, to highlight the cultural attributes and collective experiences of sports events. For example, in rural sports events, localized adaptations can be made by integrating regional folk traditions (e.g., dialect commentary, ethnic music, and dance) to enhance their cultural, ethnic, and interactive qualities. Through ritual education, these practices can strengthen students’ sense of identity and belonging to themselves, others, and society, thereby promoting their overall wellbeing.

Second, this study confirms the mediating role of perceived social support in the relationship between sports event rituals and university students’ wellbeing, highlighting the importance of enhancing students’ ability to perceive social support. In practice, parents should maintain regular communication with students via phone or video calls, particularly during key periods such as holidays or exams, to provide emotional and practical support. Universities can establish academic peer support groups, peer counseling mechanisms, or encourage students to participate in clubs and volunteer activities, thereby fostering a campus environment characterized by mutual assistance, care, and social recognition, and improving students’ perceptions of social support.

Finally, this study reveals the moderating role of physical activity levels, providing valuable guidance for differentiated interventions. Universities should continue to promote student participation in physical activity and adopt targeted strategies for different subgroups. For students with low activity levels, lightweight group activities (e.g., fitness routines, collective dances) can be implemented. Ritual elements, such as group check-ins, chanting slogans, and brief achievement sharing, can help build a sense of ritual and guide students from symbolic to sustained participation. Professional instructors can offer personalized exercise plans to avoid frustration, and “growth profiles” can track progress to boost motivation. These activities may also be integrated into campus festivals, such as group performances during sports events, to strengthen students’ identification with physical activity. For more active students, emotionally resonant rituals, like team pledges or entrance dances, can be introduced to reinforce team identity. Awards for best team atmosphere or most creative ritual can encourage innovation, and online platforms can help showcase ritual moments. Additionally, inviting retired athletes or sports celebrities to participate and share their stories can deepen students’ understanding of sportsmanship and enhance emotional engagement.

## 7 Limitations and future research

Although this study examines the impact of sports event rituals on college students’ wellbeing through perceived social support and further analyzes the moderating role of physical activity, thereby constructing a relatively systematic theoretical model, it still has several limitations that provide directions for future research.

### 7.1 Limitations and future research in sample representativeness and generalizability

The study is based on a sample of college students in Shanghai. While the sample holds a certain degree of representativeness for urban universities in China, the diversity of economic development and cultural backgrounds across regions in China limits the generalizability of the findings. Future research could expand the sample to include universities in central and western China, rural areas, or ethnic minority regions. Cross-cultural comparative studies involving students from both Eastern and Western cultural contexts would also help to explore how sports event rituals function under different cultural frameworks. Additionally, since the current sample is predominantly composed of first-year students, there may be a grade-level bias. Future studies could adopt stratified sampling to improve sample balance. Moreover, the effects of sports event rituals on other populations (e.g., working youth or older adults) deserve further exploration.

### 7.2 Limitations and future research in research design

This study employed a cross-sectional design, which limits the ability to infer dynamic causal relationships among variables. Future research could adopt longitudinal or experimental designs (e.g., pre- and post-intervention tests of changes in physical activity and wellbeing) to better assess the directionality and stability of the moderating effect of physical activity.

### 7.3 Limitations and future research in variable measurement

This study adapted sports event ritual measurements from ritual scales commonly used in tourism research, which emphasize general features such as formality, interactivity, and symbolism but may not fully capture the specific nuances of different types of sports events (e.g., large-scale multi-sport games, campus sports events, or traditional ethnic sports festivals), highlighting the need for future development of culturally and contextually tailored instruments to assess these rituals more accurately; additionally, physical activity was measured solely through self-report questionnaires, which, despite being internationally recognized standards, may introduce bias, suggesting that future research should consider incorporating wearable devices or experimental methods to minimize measurement errors.

### 7.4 Extensions of variable relationships

Guided by the issue of college students’ wellbeing, this study constructed a mediation model with perceived social support and a moderation model with physical activity to explain the effect path of sports event rituals. However, reverse or bidirectional relationships may also exist. For instance, students with higher levels of wellbeing may be more willing to engage in physical activity or more likely to perceive social support. Future research could apply structural equation modeling to validate causal assumptions or explore alternative pathways (e.g., modeling prosocial behavior as an outcome with wellbeing as a predictor), thus expanding the theoretical map of how rituals influence psychological and social behavior. In addition, future studies may consider introducing other mediating variables (e.g., self-efficacy, sense of belonging, cultural identity) or contextual moderators (e.g., cultural background, event scale, strength of group identity) to enhance the explanatory power of the theoretical model in different contexts.

## Data Availability

The original contributions presented in the study are included in the article/supplementary material, further inquiries can be directed to the corresponding author.
